# Efficiency and Sustainability in Food Supply Chains: A Systematic Analysis

**DOI:** 10.1155/sci5/3677216

**Published:** 2026-05-30

**Authors:** Abebaw Hailu Fikire, Elena Viktorovna Korchagina

**Affiliations:** ^1^ Graduate School of Service and Trade, Institute of Industrial Management, Economics and Trade, Peter the Great Saint Petersburg Polytechnic University, P.O. Box 195221, Saint Petersburg, Russia; ^2^ Department of Economics, College of Business and Economics, Debre Berhan University, P.O. Box 445, Debre Berhan, Ethiopia, dbu.edu.et; ^3^ Department of National Economics and Production Organization, Gatchina State University, Gatchina, P.O. Box 188306, Russia

**Keywords:** efficiency, food supply, supply chains, sustainability, systematic analysis

## Abstract

The inefficiency of food supply chain is a critical challenge of the global food system. Improving the efficiency and sustainability of food supply chain is essential to ensure food security and poverty reduction. The purpose of this study is to define efficiency and sustainability from the context of food supply chains and develop a conceptual framework illustrating the relationship among factors that influence efficiency and sustainability of food supply chain. The systematic review was conducted following PRISMA guidelines through encompassing 44 research articles published between 2020 and 2024 in Scopus and Web of Science indexed journals. The study revealed that distribution of annual publications has increased with some fluctuation over the reviewed period; Multidisciplinary Digital Publishing Institute was the leading publisher in the field of food supply chain. The first authors from United Kingdom and Italy were the most active principal investigators in conducting scientific research articles in the field of efficiency and sustainability of food supply chain. The findings of this research provide practical significance in clearly pointing to the directions for further study for other scholars to open new research areas, solve existing problems, and create new approaches. This review contributes to academic knowledge, policymaking, and practical implications aimed at building more efficient and sustainable food supply chains worldwide.

## 1. Introduction

Efficiency in food supply chains refers to optimizing processes to lower costs and time [1], while sustainability emphasizes reducing environmental impact and improving social responsibility across the supply chain [2]. The food supply chain comprises a few intermediaries or operates through directly linking local food producers to consumers [3]. William Petty and Adam Smith theorists primarily focused on maximizing economic results, often without explicitly addressing issues of consumption reduction [4, 5]. The Green Revolution and subsequent agricultural practices have significantly influenced the evolution of food supply systems through historical concepts of efficiency and sustainability [6]. The Green Revolution is a term used to describe the massive increase in agricultural productivity in the United States and Europe to counter hunger by boosting the production of certain crops in a relatively short period [7]. According to [8], it was estimated that 811 million individuals worldwide experienced hunger in 2020. Furthermore, Masters et al. [9] highlighted that 3 billion people struggle to afford nutritious diets due to high food prices, persistent poverty, and income inequality. The leading food‐producing countries are the United States, China, India, Brazil, and Russia, producing more than half of the world’s food supply. Africa, the Middle East, and Oceania produce only around 10% of the global output [10]. However, the concentration of global food production in a few countries has led to significant disparities in food availability, exacerbating food insecurity in vulnerable regions [11]. This inequality arises from unequal access to agricultural resources, technology, and investment, which are critical for enhancing food production and security [12]. The application of modern technology and innovation is essential to increasing the efficiency and sustainability of the food supply chain. Progress in digital technologies, including blockchain, the Internet of Things (IoT), and machine learning, has boosted operational efficiency and sustainability within food supply chains [13, 14]. The integration of these advanced technologies creates significant enhancements across multiple applications in innovative industries and transportation, leading to creative solutions [15]. Advanced technologies help manage waste, enhance traceability, and support the shift from linear to circular economies, ultimately minimizing resource use, waste, and emissions [16]. Food supply management faces external disruptions, quality management practices, and evolving consumer expectations that impact efficiency, sustainability, and food quality. Therefore, understanding these challenges is crucial for improving food supply chain operations and ensuring food security [17]. Limited studies have focused on measuring food system sustainability, integration, efficiency, food supply chains, and food loss at the regional and country levels. For instance, Adamu and Abate Alemu [18] employed the multidimensional approach method analysis to measure food system sustainability in Ethiopia. Jarzebowski et al. [19] used stochastic frontier analysis methods of analysis. Reference [20] employed statistical analysis to analyze the socioeconomic factors of global food loss. Reference [21] employed multiple reference point partially compensatory indicator to analyze sustainability of European agri‐food supply. Data envelopment analysis was employed to analyze the optimization of the sustainable food supply chain using integrative data envelopment analysis approach [22]. Reference [23] used quantitative methods specifically correlation and regression to examine the impact of agri‐food supply channels on the efficiency and links in supply chains. Reference [24] employed both decision‐making trial and evaluation laboratory and fuzzy‐Delphi method to analyze harvesting sustainability assessing Industry 4.0 in agri‐food supply chains. Improving the sustainability of food supply chains through circular economy practices a qualitative mapping approach management of environmental quality, forthcoming [25]. A novel multiobjective robust fuzzy stochastic programming model for sustainable agri‐food supply chain case study from an emerging economy [26]. Reference [27] used fuzzy decision‐making trial and evaluation laboratory method to analyze the sustainable supply chain network design for food and agricultural industries considering social and environmental impacts. There are not enough studies conducted on efficiency and sustainability of food supply chains. Thus, studying on the efficiency and sustainability of food supply chains is crucial to analyze food supply chain efficiency and sustainability to support resource utilization, decrease environmental effects, and enhance social and economic benefits. Most of the studies did not incorporate the effect of technological variables on the efficiency and sustainability of food supplies. Additionally, many studies have failed to incorporate econometric methods or regression analysis. As a result, these hinder the identification of causal relationships between efficiency measures and sustainability outcomes. Incorporating regression analysis could enhance the robustness of findings and provide valuable insights for policymakers and practitioners seeking to promote sustainable practices in various sectors. The main objective of this study is to understand the concept of efficiency and sustainability in the context of food supplies and to stress the gaps that have been identified in the existing research on food supply chains in order to emphasize the necessity of further investigation. With this in mind, it is possible to clearly point to the directions for further research for other scholars to open new areas for exploration, solve existing problems, and create new approaches. This approach helps build academic knowledge, policymaking, and practical implications to support more efficient and sustainable food supply chains worldwide. The remainder of this review is organized into five sections. Section 2 outlines the literature review results, emphasizing important findings and notable studies. Section 3 summarizes the research methodologies, offering a straightforward overview of the methods employed. In Section 4, the results are presented, along with a discussion of their implications and importance. Lastly, Section 5 wraps up the paper by summarizing the main points and suggesting directions for future research and practice.

## 2. Summary of Empirical Literature Review

Table 1 presents the empirical literature review, including authors, title of the paper, research approaches and method of analysis, and key findings of the selected studies.

**TABLE 1:** The summary of empirical literature review.

Author name and publication year	Title of paper	Research approach and methods of analysis	Findings
[18]	Measuring food system sustainability from a multidimensional perspective in Ethiopia	They used a multidimensional analytical approach method using data from 2001 to 2020.	They found that social, environmental, economic, and food and nutrition dimensions along with the overall food system sustainability index indicate a need for substantial improvement.
[19]	Assessing efficiency and integration in Poland’s food supply chain	They used the stochastic frontier analysis with panel data from 2009 to 2011.	The integration stakeholder is crucial for enhancing performance, reducing costs, and improving overall efficiency.
[20]	Socioeconomic factors of global food loss	The study used statistical analysis. The time series data were collected from 81 countries ranging from 2000 until 2020.	They found that high gross national income per capita, high agricultural employment, access to electricity in rural areas, and a high export volume index are significantly associated with low food loss.
[21]	Sustainability of the European agri‐food supply chain of the European Union countries through a multicriteria analysis	The study used a multiple reference point partially, compensatory indicator based on a set of 50 indicators from 2011 to 2019	They found that economic, social, and environmental dimensions are crucial factors for evaluating the performance of the agri‐food supply chains across European countries.
[22]	Optimization of the sustainable food supply chain with an integrative data envelopment analysis approach.	The researchers employed an integrative data envelopment analysis approach.	The study results can provide a strategic choice for critical decision makers in the food supply chain network under sustainability consideration.
[23]	Impact of agri‐food supply channels on efficiency and links in supply chains	Quantitative methods based on correlation and regression analysis were applied using Kazakh statistical data from 2008 to 2022.	They found that the increase in the efficiency of supply channels in the commodity distribution system leads to a decrease in the coefficient in commodity movement and inventory availability. Therefore, it is necessary to improve supply chain efficiency at each link and reduce the share of retail trade in gross turnover.
[24]	Harvesting sustainability: Assessing Industry 4.0 in agri‐food supply chains	The study used decision‐making trial and evaluation laboratory and fuzzy‐Delphi method	The study found that market competitiveness knowledge and skill development, resource efficiency, and technologies are essential for increasing the marketability of agricultural products.
[25]	Improving the sustainability of food supply chains through circular economy practices	They used a qualitative approach grounded in circular economic perspectives.	They found mapped food waste flows and industrial linkages along the supply chain.
[27]	Modeling the sustainable supply chain network design for food‐agricultural industries considering social and environmental impacts	They used the fuzzy decision‐making trial and evaluation laboratory.	They found that the most effective were the financial dimension, the use of high technology in production, and the presentation of various citrus forms through intermediate and conversion industries.
This study	Efficiency and Sustainability in Food Supply Chains: A Systematic Analysis	Systematic literature review	The efficiency and sustainability of food supply chain can be defined as the optimal flow of food products from the production to consumption stages, with minimal losses, costs, and delays, utilizing available economic, social, and technological resources, without harming future generation.

### 2.1. Conceptual Framework on Efficiency and Sustainability in Food Supply Chain

**FIGURE 1:**
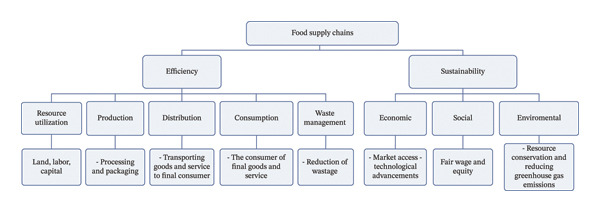
Conceptual framework of efficiency and sustainability in food supply chains. Source: adapted from [2, 8] modified with empirical literature.

Figure 1 indicates that efficiency and sustainability in food supplies integrate multiple dimensions, highlighting the interdependencies between resource utilization, production, distribution, consumption, waste management, environmental impact, economic viability, and social equity. This framework provides a holistic view of the interdependencies between food supply chain efficiency and sustainability, fostering a more integrated approach to addressing food system challenges and achieving efficient and sustainable food supplies across the globe [27]. Therefore, policymakers, businesses, and consumers must collaborate to create robust governing structure that encourages innovation and investment in sustainable food practices, ensuring that food supply chains are efficient in production, equitable, and environmentally sound.

## 3. Methodology

### 3.1. Search Strategies and Data Collection Process

This systematic article review aims to explore the concepts of efficiency and sustainability in the context of food supplies and provide a comprehensive review of the structure and networks of food supply on a global scale. This offer provides a comprehensive perspective and several insights for additional research in diverse new business scenarios. A systematic literature review provides a robust methodology for objectively analyzing evidence, effectively managing numerous scholarly publications, and creating a sophisticated framework for research topics. It enhances the overall evaluation of current knowledge and identifies gaps for future research opportunities. This study collectively emphasizes the importance of identifying research gaps and employing appropriate methodologies to address them in various fields. Systematic literature reviews follow a structured method to synthesize evidence on a specific research question. Standard procedures include defining the research question and eligibility criteria, conducting a comprehensive literature search, selecting relevant studies, extracting data, assessing study quality, and synthesizing findings. This methodology enables researchers to integrate existing knowledge, identify gaps, and propose future research agendas in efficient and sustainable food supply chains. A systematic review should begin with a peer‐reviewed procedure and clearly state its methodology to established reporting guidelines of Preferred Reporting Items for Systematic Reviews and Meta‐Analyses (PRISMA) [28]. This study used PRISMA guideline to identify and screen the literature for inclusion. Table A1 presents the PRISMA checklist, detailing the reporting guidelines followed in this study.

#### 3.1.1. Year‐Based Classification of Publications

The first step is to select the time frame and find the research articles published on the efficiency and sustainability of food supply chains between 2020 and 2024.

#### 3.1.2. Categorization of Journal Name Based on the Papers

The published articles were categorized by journal names based on number of published articles on the efficiency and sustainability of food supply chains.

#### 3.1.3. Categorization Based on Database Systems

The research articles were selected from Scopus and Web of Science databases. They were written in English and included the following subject areas: social science indexing, business and economics, economics and econometrics, and business management and accounting. Most of the articles were collected from the Scopus database. Scopus is useful for investigating complex research topics. It has more journals than the Web of Science, 96.61% of which are indexed, and its larger coverage allows researchers to access a broader range of articles [29]. This study further confirms that the Scopus database provides a better coverage than Web of Science.

#### 3.1.4. Keywords Used for Article Selection

This review used the keywords to select the published articles from Scopus and Web of Science databases: “efficiency AND sustainability AND food supply chains” applied to articles, abstract and keywords.

#### 3.1.5. The Systematic Review Process for the Classification of Academic Publications

In the “identification” stage, an initial pool of records was gathered from Scopus and Web of Science databases. Specifically, 1232 records were identified, including 643 records from Scopus and 589 records from Web of Science. Before further analysis, a significant number of records (1184) were automatically removed because they were deemed ineligible by automation tools, demonstrating an effective preliminary filter. The number of research articles was further reduced by including only academic journals in the review. The research papers excluded from the review are fell outside the subject areas and were not written in English, excluded document types reviews, book chapters, conference papers, books, editorials, and conference reviews. After excluding these articles, 44 remained in Scopus and 4 in the Web of Science as automatically filtered from the databases.

**FIGURE 2:**
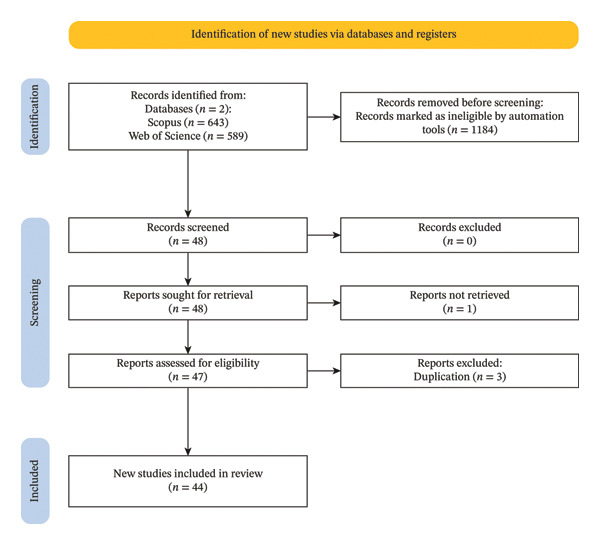
PRISMA flow diagram of the study selection process. Source: [30].

In the “screening” stage, the records were carefully evaluated to remove irrelevant papers and duplicates based on their titles. After this refinement, 48 records remained from both databases. An additional exclusion process was conducted in which one record was removed one record was removed due to open‐access restrictions. The remaining 47 reports were evaluated for “eligibility” in the following step. During this assessment, 3 records were excluded due to duplication problems. Finally, 44 high‐quality and relevant studies were included the final body of the reviewed literature. Figure 2 presents the PRISMA flow diagram illustrating the systematic literature review article selection process.

#### 3.1.6. Classification Based on Research Approach and Methods of Analysis

The categorization of the research papers based on research approaches and methods is crucial for organizing and conducting research across various disciplines. Therefore, this study classified a research article used to analyze the efficiency and sustainability of food supply chains.

#### 3.1.7. Classification of Published Research Papers by Publisher

Many major publishers are identified in the paper, such as Elsevier, Wiley‐Blackwell, Springer, Taylor, and Multidisciplinary Digital Publishing Institute (MDPI), while others are published in peer‐reviewed journals that contribute to prior knowledge in various disciplines. The category of publications plays a significant role in the creation and availability of multiple publications in the market.

#### 3.1.8. Geographical Area and Region

The review categorized publications based on the country of the first authors. It is essential for understanding the national research trends, collaborative networks, funding availability, and the global distribution of academic output. Therefore, analyzing the first authors’ countries helps the researchers identify the potential gaps and foster international partnerships to address pressing global challenges. Additionally, the research publications can be categorized by region to understand better the focus and distribution of scientific inquiry across different geographical areas. Analyzing research outputs at regional level is very important as it enables stakeholders to identify collaborative opportunities, funding needs, and strategic priorities to enhance the impact of global research.

## 4. Result and Discussion

The following sections present, discuss, and analyze all the identified papers, detailing their various aspects and features.

### 4.1. Annual Distribution of Publications

**FIGURE 3:**
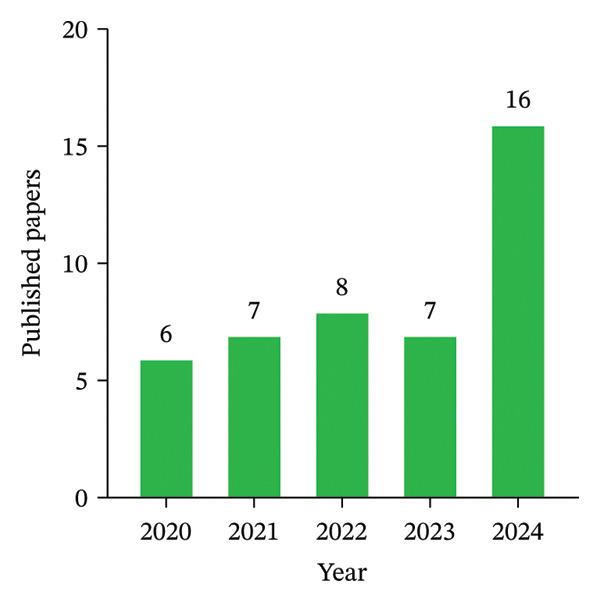
Annual distribution of publications per year.

Figure 3 illustrates the annual distributions of publications concerning the food supply chains from 2020 to 2024. In 2020, there were approximately 6 journal publications, which slightly increased to 7 in 2021. These upward trends continued in 2022, with a count of 8. Although a slight decline with 7 publications was observed in 2023, the field saw a significant upsurge in 2024 with 16 publications. Overall, the data indicate an upward publication trend with some fluctuation in 2023 on the efficiency and sustainability of food supply chains.

### 4.2. Categorization of Publications by Journal Source

**FIGURE 4:**
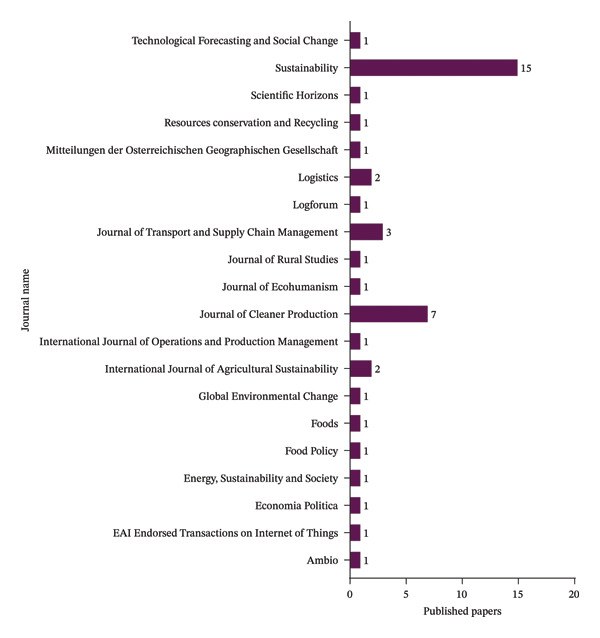
Distribution of publication by journal.

Figure 4 shows the distribution of academic articles by journal sources. The analysis indicates that Sustainability and Journal of Cleaner Production journals led the field with 15 and 7 papers, respectively. Journal of Transport and Supply Chain Management contributed three research articles, while International Journal of Agricultural Sustainability and Logistics published two articles each. The remaining journals contributed fragmented articles distributed with one paper per journal. This pattern suggested diverse but less intensive publication records across several journals, demonstrating a wide range of research topics and contributions. The low numbers across numerous journals may indicate newly developing research areas, specialist themes, or publications with tougher publication standards.

### 4.3. Distribution of Publications by Publisher

**FIGURE 5:**
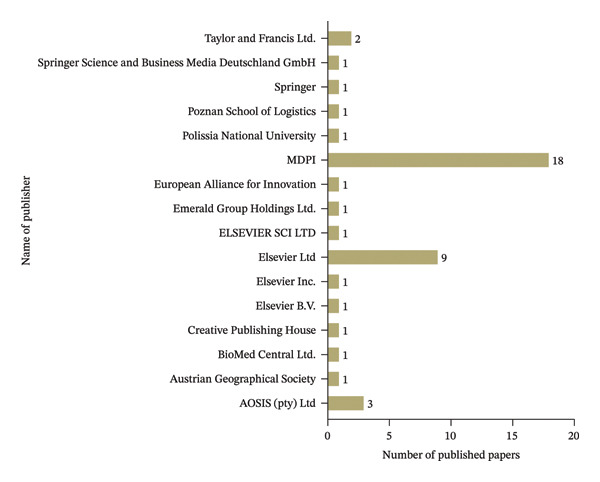
Distribution of publications by publisher.

Figure 5 presents the categorization of published articles on food supply by publisher. Notably, MDPI leads with 18 publications, demonstrating its prominent role in spreading research on efficiency and sustainability of food supply chain. This is followed by Elsevier Ltd and Tayler and Francis Ltd with nine and two articles, respectively, establishing them as a significant contributor to this research field. Conversely, a wide array of publishers including AOSIS (pty) Ltd, BioMed Central Ltd, Creative Publishing House, Springer, Elsevier B.V, Elsevier Inc., Elsevier SCILTD, Emerald Group Holding Ltd, European Alliance for Innovation, Polissia National University, Poznan School of logistics, Austrian Geographical Society, Springer International Publishing, and Frontiers Media SA each contributed a single publication. Despite their minor contributions, these publishers bring diversity to food supply chains.

### 4.4. Distribution of Publications by Database Sources

**FIGURE 6:**
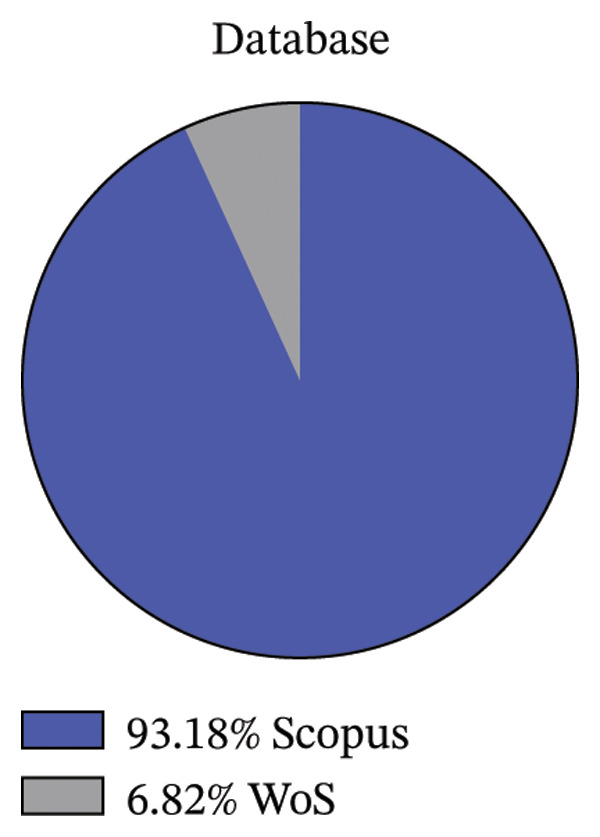
Distribution of publications by indexing database.

Figure 6 shows the distribution of publications across databases, specifically comparing Scopus and Web of Science. The data reveal that the vast majority of publications (93%) are indexed in Scopus, establishing it as the predominant indexing service for research in this field. This dominance suggests that Scopus offers higher visibility and possibly a preference for researchers focusing on efficiency and sustainability of food supply chains. In contrast, Web of Science indexes 7% of articles. The share is notably lower than Scopus but still significant and demonstrates the importance and hope of research expansion by many indexing services.

### 4.5. Categorization of Publications by Country of First Author

**FIGURE 7:**
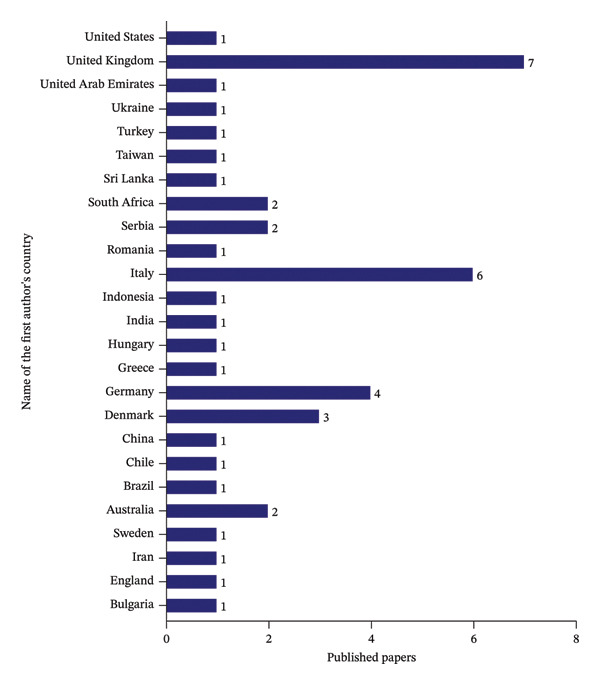
Categorization of publications by national affiliation of the first author.

Figure 7 indicates the geographical distribution of publications based on the first author’s country of affiliation. The United Kingdom and Italy emerge as significant contributors, accounting for 7 and 6 of the total papers, respectively. These figures signify a robust infrastructure and highly focused efficiency and sustainability of the food supply chains within these nations. Following these, significant contributions are also observed from Germany (4) and Denmark (3). Furthermore, Serbia, South Africa, and Australia each contributed two publications. The data indicate that these countries provide moderate contributions with a lesser extent than the top contributor’s country. However, United States, United Kingdom, United Arab Emirates, Ukraine, Turkey, Taiwan, Sri Lanka, Romania, Indonesia, India, Hungary, Greece, China, Chile, Brazil, Sweden, Iran, England, and Bulgaria each contributed one publication. While these individual contributions are smaller in scale, they collectively represent diverse worldwide research efforts in food supply efficiency and sustainability.

### 4.6. Distribution of Publications by Research Method and Approaches

**FIGURE 8:**
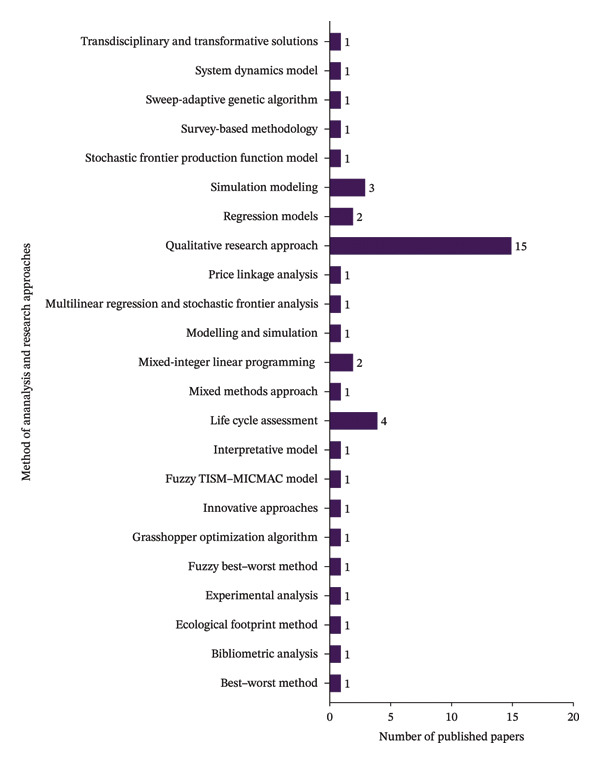
Categorization based on research methods and approaches.

Figure 8 illustrates the categories of research articles by methodological approach and analytical framework, highlighting diverse techniques employed in food supply chain studies. The data demonstrate a predilection for extensive context‐specific inquiries in this research area, which could provide in‐depth insights and practical applications on these fields. Specifically, qualitative research approach including interviews, case studies, focus groups and narratively described in words also show a significant representation with 15 publications. This substantial qualitative underscore the importance of narrative data in understanding and address the complexities of food supply chain efficiency and sustainability. Whereas the life cycle assessment has 4 research articles, simulation modeling, mixed‐integer linear programming and regression model appeared three and two publications, respectively. The following methods are each represented by one publication: best–worst method, bibliometric analysis, ecological footprint method, experimental analysis, fuzzy best–worst method, grasshopper optimization algorithm, innovative approaches, integrated total interpretive structural modeling and fuzzy, matrix impact cross‐reference multiplication applied to a classification analysis, interpretative model, mixed‐methods approach, modeling and simulation, multilinear regression and stochastic frontier analysis, price linkage analysis, stochastic frontier production function model, survey‐based methodology, sweep‐adaptive genetic algorithm, system dynamics model, and transdisciplinary and transformative solutions. This result suggests an interdisciplinary and multifaceted approach required to address food sustainability and efficiency issues. Each method brings a unique perspective and set of tools, from qualitative to quantitative and from theoretical frameworks to empirical analyses.

### 4.7. Keywords Used in Efficiency and Sustainability of Food Supply Chains

**FIGURE 9:**
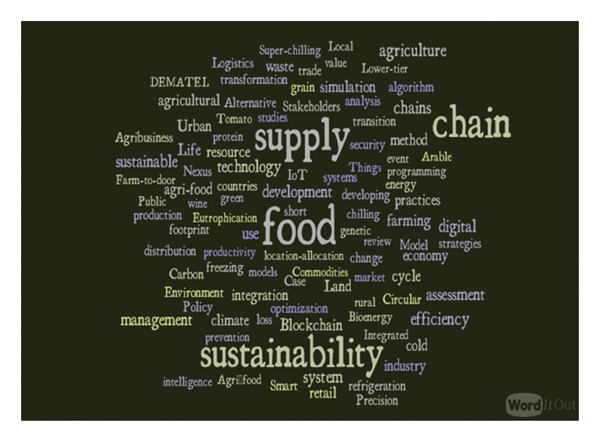
Keywords in efficiency and sustainability of food supply chains.

Figure 9 shows the keywords related to efficiency and sustainability of food supply chains. The terms “food,” “supply,” “chain,” and “sustainability” are the most frequently used keywords. This indicates that the existing literature is highly concentrated on core concepts of sustainable food supply chain, while addressing environmental, economic, and social sustainability challenges. “Efficiency,” “waste,” “integration,” and “sustainable” are also significant keywords, which are concerned about optimizing the food supply chains. “Blockchain,” “digital,” “simulation,” and “IoT” reference the growth of technology in modern food system. Additionally, “agriculture, “farming,” “climate,” “resource,” and “environment” highlight the connection between food production and ecological factors. This further suggests that scholars are prioritizing integrated approaches that link food production, distribution, and consumption within a sustainability framework.

### 4.8. Categorization of Research Publications by Region

**FIGURE 10:**
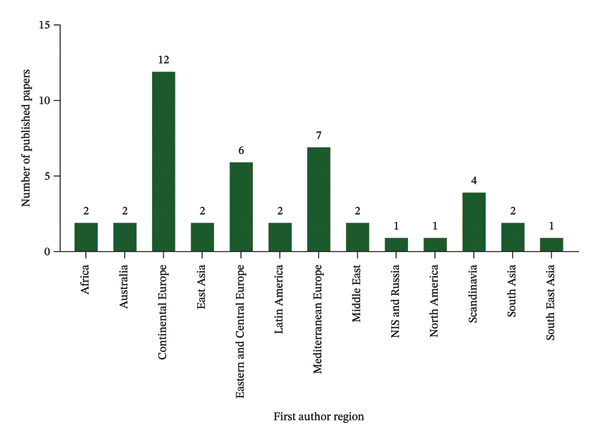
Categorization of research publications by region.

Figure 10 depicts the proportional contributions of various locations to the total by continent. Continental Europe dominated the survey, accounting for 12 of the total published articles. This makes Continental Europe as the most important contributor among the regions. Following this, Mediterranean Europe has the second‐largest contribution with 7 articles, indicating that it is also a significant contributor. The Eastern and Central Europe is close behind accounting for 6 articles. Scandinavia contributed 4 articles. Other major regions include Africa, Australia, East Asia, Latin America, Middle East, and South Asia, each contributing two publications. This indicates a considerable and equal publication contribution from these regions. Meanwhile, NIS and Russia, North America, and South Asia each contribute one publication. These regions collectively account for less of the entire distribution. Despite their smaller individual contributions, they collectively contribute to the overall distribution.

## 5. Conclusion and Future Research Directions

Food supply chain faces critical socioeconomic and technological challenges that limit the networked food product flows. Therefore, addressing the inefficiency and sustainability of food supply chain required multidimensional approaches. This study provides an exhaustive investigation of defining efficiency and sustainability ideas in the context of food supplies. This review develops a conceptual framework that explains the structure relationship among efficiency, sustainability, and food supply chain networks across the globe. This study used PRISMA guidelines to include the relevant research articles in the study. In this study, inclusion and exclusion criteria were employed to ensure the relevance and quality of the research. This study used standardized systematic approach, time zone and publication selection, systematic review process for classification of academic publications, research method and approach, categorization of academic journals, and geographical area and region. It is common to develop a systematic review and clearly explain the procedure for selecting research articles from different databases. These reflect a highly selective and structured approach to ensure that only quality and pertinent studies constitute the final body of researched literature. The review shows an annual increase in journal publications from 2020 to 2024. This implies a growing recognition of the importance of food supply, and there is a global concern attracting the attention of numerous experts who want to address inefficiency and fragmented food supply chains. This review found that Sustainability and the Journal of Cleaner Production were leaders in reporting on food supply chain issues. MDPI also leads in publishing research articles on food supplies. This also confirms that the Scopus database indexing system is more crucial in accessing research articles in food supply chains than the Web of Science indexing system. The United Kingdom and Italy’s first authors are the leading role in researching food supplies more than other countries. This indicates that United Kingdom and Italy provide more significant research budgets and more emphasis on improving efficiency and sustainability of food supply chains. Besides, Continental Europe was the prominent region in food supplies, followed by Mediterranean Europe. The systematic literature review reveals a wide range of intentions to undertake research on the food supply chain in various parts of the world. Future studies should focus on enhancing the efficiency and sustainability of food supply chains through widely integrating digital technology in the food system. Furthermore, this study invites researchers to conduct on the global multisectoral development of food supply chains by using econometrics model to examine the causal relationships between efficiency measures and sustainability outcomes.

## Author Contributions

Conceptualization, Abebaw Hailu Fikire and Elena Viktorovna Korchagina; methodology, Abebaw Hailu Fikire; software, Abebaw Hailu Fikire; validation, Abebaw Hailu Fikire and Elena Viktorovna Korchagina; formal analysis, Abebaw Hailu Fikire; investigation, Abebaw Hailu Fikire; resources, Abebaw Hailu Fikire; data curation, Abebaw Hailu Fikire; writing–original draft preparation, Abebaw Hailu Fikire; writing–review and editing, Elena Viktorovna Korchagina; visualization, Abebaw Hailu Fikire; and supervision, Elena Viktorovna Korchagina.

## Funding

This study received no funding.

## Disclosure

All authors have read and agreed to publish this version of the manuscript.

## Ethics Statement

The authors have nothing to report.

## Consent

The authors have nothing to report.

## Conflicts of Interest

The authors declare no conflicts of interest.

## Data Availability

The authors have nothing to report.

## Appendix A PRISMA 2020 Checklist

**Table A1:** PRISMA checklist reporting guidelines.

Section and topic	Item #	Checklist item	Location/page # where item is reported
*Title*			
Title	1	Identify the report as a systematic review.	1

*Abstract*			
Abstract	2	See the PRISMA 2020 for abstract checklist.	1

*Introduction*			
Rationale	3	Describe the rationale for the review in the context of existing knowledge.	3–5

Objectives	4	Provide an explicit statement of the objective(s) or question(s) the review addresses.	5

*Methods*			
Eligibility criteria	5	Specify the inclusion and exclusion criteria for the review and how studies were grouped for the syntheses.	11

Information sources	6	Specify all databases, registers, websites, organizations, reference lists, and other sources searched or consulted to identify studies. Specify the date when each source was last searched or consulted.	11

Search strategy	7	Present the full search strategies for all databases, registers, and websites, including any filters and limits used.	11

Selection process	8	Specify the methods used to decide whether a study met the inclusion criteria of the review, including how many reviewers screened each record and each report retrieved, whether they worked independently, and if applicable, details of automation tools used in the process.	12

Data collection process	9	Specify the methods used to collect data from reports, including how many reviewers collected data from each report, whether they worked independently, any processes for obtaining or confirming data from study investigators, and if applicable, details of automation tools used in the process.	10–13

Data items	10a	List and define all outcomes for which data were sought. Specify whether all results that were compatible with each outcome domain in each study were sought (e.g., for all measures, time points, and analyses), and if not, the methods used to decide which results to collect.	NA
	10b	List and define all other variables for which data were sought (e.g., participant and intervention characteristics and funding sources). Describe any assumptions made about any missing or unclear information.	NA

Study risk of bias assessment	11	Specify the methods used to assess risk of bias in the included studies, including details of the tool(s) used, how many reviewers assessed each study and whether they worked independently, and if applicable, details of automation tools used in the process.	NA

Effect measures	12	Specify for each outcome the effect measure(s) (e.g., risk ratio and mean difference) used in the synthesis or presentation of results.	NA

Synthesis methods	13a	Describe the processes used to decide which studies were eligible for each synthesis (e.g., tabulating the study intervention characteristics and comparing against the planned groups for each synthesis (item #5)).	10–13
	13b	Describe any methods required to prepare the data for presentation or synthesis, such as handling of missing summary statistics, or data conversions.	13–21
	13c	Describe any methods used to tabulate or visually display results of individual studies and syntheses.	NA
	13d	Describe any methods used to synthesize results and provide a rationale for the choice(s). If meta‐analysis was performed, describe the model(s), method(s) to identify the presence and extent of statistical heterogeneity, and software package(s) used.	NA
	13e	Describe any methods used to explore possible causes of heterogeneity among study results (e.g., subgroup analysis and meta‐regression).	NA
	13f	Describe any sensitivity analyses conducted to assess robustness of the synthesized results.	NA

Reporting bias assessment	14	Describe any methods used to assess risk of bias due to missing results in a synthesis (arising from reporting biases).	NA

Certainty assessment	15	Describe any methods used to assess certainty (or confidence) in the body of evidence for an outcome.	NA

*Results*			
Study selection	16a	Describe the results of the search and selection process, from the number of records identified in the search to the number of studies included in the review, ideally using a flow diagram.	12–21
	16b	Cite studies that might appear to meet the inclusion criteria, but which were excluded, and explain why they were excluded.	12

Study characteristics	17	Cite each included study and present its characteristics.	NA

Risk of bias in studies	18	Present assessments of risk of bias for each included study.	NA

Results of individual studies	19	For all outcomes, present, for each study: (a) summary statistics for each group (where appropriate) and (b) an effect estimate and its precision (e.g., confidence/credible interval), ideally using structured tables or plots.	NA

Results of syntheses	20a	For each synthesis, briefly summarize the characteristics and risk of bias among contributing studies.	NA
	20b	Present results of all statistical syntheses conducted. If meta‐analysis was done, present for each the summary estimate and its precision (e.g., confidence/credible interval) and measures of statistical heterogeneity. If comparing groups, describe the direction of the effect.	NA
	20c	Present results of all investigations of possible causes of heterogeneity among study results.	NA
	20d	Present results of all sensitivity analyses conducted to assess the robustness of the synthesized results.	NA

Reporting biases	21	Present assessments of risk of bias due to missing results (arising from reporting biases) for each synthesis assessed.	NA

Certainty of evidence	22	Present assessments of certainty (or confidence) in the body of evidence for each outcome assessed.	NA

*Discussion*			
Discussion	23a	Provide a general interpretation of the results in the context of other evidence.	13–21
	23b	Discuss any limitations of the evidence included in the review.	?
	23c	Discuss any limitations of the review processes used.	?
	23d	Discuss implications of the results for practice, policy, and future research.	22

*Other information*			
Registration and protocol	24a	Provide registration information for the review, including register name and registration number, or state that the review was not registered.	NA
	24b	Indicate where the review protocol can be accessed, or state that a protocol was not prepared.	No
	24c	Describe and explain any amendments to information provided at registration or in the protocol.	No

Support	25	Describe sources of financial or nonfinancial support for the review, and the role of the funders or sponsors in the review.	22

Competing interests	26	Declare any competing interests of review authors.	22

Availability of data, code, and other materials	27	Report which of the following are publicly available and where they can be found: template data collection forms; data extracted from included studies; data used for all analyses; analytic code; and any other materials used in the review.	22
